# Assessment of Gastroesophageal Reflux Disease in Patients Exhibiting Otolaryngology Symptoms at a Tertiary Care Hospital

**DOI:** 10.7759/cureus.99142

**Published:** 2025-12-13

**Authors:** Thamer Almasoudi, Samar S Alsifri, Renad Alosaimi, Suhail H Alrudaini, Samar M Altoukhi, Raseel Alshehri, Abdullah S Alfarsi, Danah S Alhajjaji, Raghad O Al-Masoudi

**Affiliations:** 1 Department of Gastroenterology, King Fahad Armed Forces Hospital, Jeddah, SAU; 2 College of Medicine and Surgery, Umm Al-Qura University, Makkah, SAU; 3 Faculty of Medicine, King Abdulaziz University, Jeddah, SAU; 4 College of Medicine, University of Jeddah, Jeddah, SAU; 5 Department of Internal Medicine, King Fahad Armed Forces Hospital, Jeddah, SAU

**Keywords:** diabetes mellitus, extraesophageal symptoms, gastroesophageal reflux disease, gerd los angeles classification, multidisciplinary care, otolaryngology manifestations, proton pump inhibitor, shortness of breath (sob)

## Abstract

Background

Gastroesophageal reflux disease (GERD) is characterized by repeated episodes of heartburn sensation or regurgitation; it can also present as extraesophageal symptoms, which include dysphagia and hoarseness alongside throat clearing. Patients frequently present with atypical GERD symptoms, which trigger admission to otolaryngology (ENT) practices; however, this often results in patients missing the opportunity for proper diagnosis and treatment by gastroenterologists.

Objectives

This study aims to analyze the frequency of ENT complaints among GERD patients referred to gastroenterology from ENT and to determine their response to proton pump inhibitor (PPI) therapy based on structured clinic follow-up regarding symptomatic improvement following initiation of the regimen.

Methods

This retrospective record review study was conducted at a tertiary hospital in Saudi Arabia, between January 1, 2018, and December 31, 2023. A total of 263 adult patients diagnosed with GERD and referred for ENT-related symptoms were included using consecutive sampling. Inclusion criteria were age ≥17 years, confirmed GERD based on either clinical or endoscopic findings via the Los Angeles classification (LA) grading system, and documented ENT complaints (e.g., sore throat, dysphagia, hoarseness, halitosis). Exclusion criteria were incomplete records, chronic pulmonary disease, or a history of neck/glottic surgery, radiotherapy, or malignancy. Data were collected from hospital records and analyzed using IBM SPSS Statistics for Windows, version 26 (IBM Corp., Armonk, New York, United States). The chi-square test was used to assess associations, with p<0.05 considered significant.

Results

The participants had a mean age of 50.7 years ± 20.2 years, with 168 female patients (63.9%) in the sample. The majority of patients (78.3%) exhibited GERD LA grade A, whereas dysphagia in 83 patients (31.6%) and sore throat in 74 patients (28.1%) were the most frequent ENT symptoms among patients. GERD LA grade C was significantly correlated with halitosis development (P<0.001). The treatment benefit reached 230 patients (87.5%), but halitosis development (p<0.001), snoring (p=0.01), and shortness of breath (p=0.036) were associated with unfavorable treatment responses.

Conclusions

Patients with ENT symptoms may have GERD even in the absence of typical reflux symptoms. Symptoms of halitosis and snoring indicate advanced or treatment-resistant GERD. Accurate diagnosis and effective management depend on tight collaboration between multiple medical disciplines.

## Introduction

Gastroesophageal reflux disease (GERD) is characterized by repeated episodes of heartburn sensation or regurgitation; it can generate extraesophageal symptoms such as hoarseness, globus, and throat clearing, or a patient may be asymptomatic [1-5]. Many of these patients are seen at otorhinolaryngologic (ENT) clinics, where they are frequently treated with proton pump inhibitors (PPI) on the basis solely of the results of transnasal flexible laryngoscopy and the presence of symptoms. Similarly, most gastroenterologists recommend starting in patients with pharmacological trials such as a PPI rather than objective tests [6-8].

Upper gastrointestinal endoscopy and esophageal (typical) symptoms have been repeatedly shown to be ineffective in diagnosing GERD [9,10]. Extraesophageal symptoms may be even more misleading because they may be caused by a range of conditions other than GERD. Assessment should include evaluation of esophageal motility (to rule out achalasia), accurate placement of the pH catheter 5 cm above the lower esophageal sphincter (as determined by manometry), and determination of the presence and proximal extent of abnormal reflux in patients with ENT symptoms thought to be caused by GERD [11-14]. Assessing GERD in patients referred to gastroenterology for ENT symptoms in tertiary care facilities is a complicated task. The management needs a comprehensive approach because GERD shows inconsistent typical symptoms during diagnosis [15-19]. Multiple diagnostic approaches involving questionnaires, endoscopy, and pH monitoring should be employed when identifying and associating GERD symptoms with ENT conditions such as chronic cough, hoarseness, and throat clearing [20,21].

The assessment and management of patients with GERD requires joint medical collaboration between gastroenterologists and otolaryngologists, who must consider treatment options, including lifestyle modifications or medication and possible surgical intervention [22,23]. Effective long-term management, especially with slow disease progression, requires a deep understanding of patient interactions to achieve better patient care outcomes in tertiary care settings. New research investigating GERD manifestations among patients referred to gastroenterology from ENT is inconclusive in both the Middle East and Saudi Arabia. Therefore, this study aimed to investigate how often ENT-related symptoms occur in GERD patients and to assess their clinical response to PPI therapy through structured follow-up focused on symptomatic improvement after treatment initiation.

## Materials and methods

This was a retrospective record review study conducted at the King Fahad Armed Forces Hospital (KFAFH), a tertiary referral center in Jeddah, Saudi Arabia. The study covered a six-year period from January 1, 2018, to December 31, 2023. The study was approved by the Research Ethics Committee of Armed Forces Hospitals-Jeddah (Reference: REC 675; Project application number: 2024-33).

Study population and sampling technique

All eligible patients were identified through the hospital’s electronic medical record system. A consecutive sampling method was employed to include every case that fulfilled the eligibility criteria within the specified study period.

Inclusion criteria encompassed patients aged 17 years and older who were diagnosed with GERD, either clinically or endoscopically, and had documented ENT symptoms, including sore throat, dysphagia, hoarseness, halitosis, or chronic cough. Exclusion criteria comprised incomplete or missing medical records, a history of chronic pulmonary or tracheobronchial disease, previous neck or glottic surgery, prior radiotherapy, or any malignancy involving the upper aerodigestive tract. 

Data collection

Data were extracted using a standardized collection form using information obtained from the patient's electronic medical records, which are available in the KFAFH computerized database. Variables included demographic information (age and sex), clinical comorbidities such as diabetes mellitus and hypertension, and detailed ENT symptom profiles, including symptom type and duration. Endoscopic findings were recorded as either normal or abnormal, with reflux severity classified according to the Los Angeles (LA) grading system. This classification describes the severity of the condition using the following grades: Grade A: ≥1 esophageal mucosal breaks <5 mm long, Grade B: ≥1 mucosal breaks >5 mm but with continuity across mucosal folds, Grade C: Continuous mucosal breaks between the tops of ≥2 mucosal folds but involving <75% of the esophageal circumference and Grade D: Mucosal breaks involving >75% of the esophageal sphincter [24]. Treatment response was assessed based on improvement or worsening following PPI therapy.

Statistical analysis

Microsoft Excel (Microsoft Corporation, Redmond, Washington, United States) was used for data entry, and data analysis was performed using IBM SPSS Statistics for Windows, Version 26.0 (Released 2019; IBM Corp., Armonk, New York, United States). Continuous variables were expressed as mean ± standard deviation (SD), whereas categorical variables were summarized as frequencies and percentages. Associations between categorical variables were evaluated using the chi-square test or Fisher’s exact test, as appropriate. A p-value < 0.05 was considered statistically significant with a confidence interval of 95%.

## Results

Among the 263 patients included in the studies, 138 were aged ≤ 50 years (52.4%), with a mean age of 50.71 ± 20.22 years. Among them, 168 were female (63.9%). We identified that 119 patients (45.2%) had chronic diseases, with diabetes mellitus being the most prevalent, affecting 73 individuals (61.3%). Hypertension was also common, present in 68 patients (57.1%). Table 1 shows the sociodemographic and clinical characteristics of the participants.

**Table 1:** Distribution of the studied patients according to their demographic characteristics and chronic diseases (N=263)

Variable	Frequency	Percentage
Age (years)		
≤50	138	52.4
>50	125	47.6
Sex		
Female	168	63.9
Male	95	36.1
Chronic diseases		
No	144	54.8
Yes	119	45.2
If having a chronic disease, specify (n=119)		
Diabetes mellitus	73	61.3
Hypertension	68	57.1
Thyroid disorders	27	22.6
Heart disorders	8	6.7
Renal disorders	3	2.5
Respiratory disorders	8	6.7
Other	15	12.6

Table 2 shows that a substantial number of patients (76.8%) exhibited normal results during endoscopic evaluations. Among the participants, a significant majority (n=193, 73.3%) reported experiencing ENT symptoms for durations exceeding two months, with an average symptom duration of 2.72 months ± 1.15 months. The classification of GERD severity showed that most patients (n=206, 78.3%) were categorized as LA grade A, indicating milder disease. In contrast, 56 patients (21.3%) were classified as LA grade B, and only one patient (0.4%) fell into the LA grade C category, representing more severe disease. Encouragingly, the treatment results indicated that a significant proportion of patients (n=230, 87.5%) experienced improvement in their symptoms.

**Table 2:** Distribution of studied patients according to endoscopy report, ENT symptoms duration (months), GERD classification, and response to treatment (N=263) NA: not available; GERD: gastroesophageal reflux disease

Variable	Frequency	Percentage
Endoscopy report		
Normal	202	76.8
Abnormal findings	61	23.2
Symptoms duration (months)		
1-2	64	24.3
>2	193	73.3
NA	6	2.4
GERD classification		
LA grade A	206	78.3
LA grade B	56	21.3
LA grade C	1	0.4
Response to treatment		
Getting worse	4	1.5
Improve	230	87.5
No improvement	29	11

**Figure 1:**
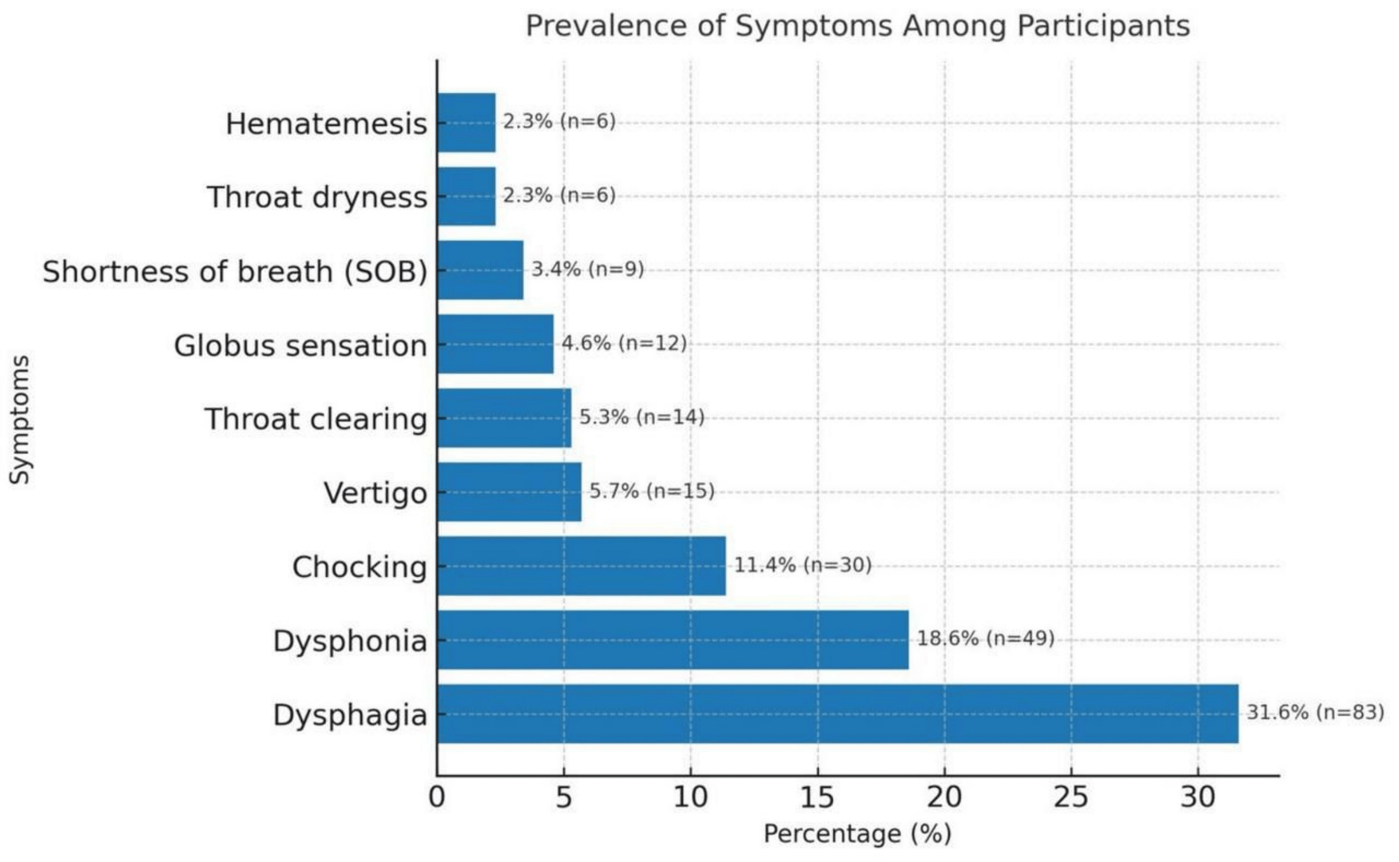
Distribution of ENT symptoms among GERD patients GERD: gastroesophageal reflux disease

Figure 1 illustrates the prevalence of various ENT symptoms observed in patients diagnosed with GERD. The most frequently reported symptom was dysphagia, affecting 83 patients (31.6%). This was closely followed by sore throat, which was reported by 74 patients (28.1%). Dysphonia was noted in 49 patients (18.6%), while cough was experienced by 34 patients (12.9%). Additionally, 30 patients (11.4%) reported a sensation of choking.

Table 3 presents data indicating that patients classified with GERD as LA grade C exhibited a significantly higher prevalence of halitosis (p <0.05). Conversely, the analysis revealed that there was a nonsignificant relationship between the classification of GERD and other ENT symptoms (p ≥0.05).

**Table 3:** Relationships between GERD classification and ENT symptoms (N=263) GERD: gastroesophageal reflux disease; LA: Los Angeles

Variable	GERD classification			χ2	p value
	LA grade A, n (%)	LA grade B, n (%)	LA grade C, n (%)		
Dysphagia	60 (29.1)	23 (41.1)	0 (0.0)	3.37	0.185
Sore throat	61 (29.6)	13 (23.2)	0 (0.0)	1.28	0.526
Dysphonia	39 (18.9)	10 (17.9)	0 (0.0)	0.26	0.877
Cough	27 (13.1)	7 (12.5)	0 (0.0)	0.16	0.922
Chocking	25 (12.1)	5 (8.9)	0 (0.0)	0.57	0.749
Tinnitus	11 (5.3)	5 (8.9)	0 (0.0)	1.05	0.589
Vertigo	13 (6.3)	2 (3.6)	0 (0.0)	0.67	0.714
Odynophagia	12 (5.8)	3 (5.4)	0 (0.0)	0.07	0.961
Throat clearing	14 (6.8)	0 (0.0)	0 (0.0)	4.09	0.129
Halitosis	10 (4.9)	2 (3.6)	1 (100)	19.45	<0.001
Globus sensation	9 (4.4)	3 (5.4)	0 (0.0)	0.14	0.929
Snoring	10 (4.9)	0 (0.0)	0 (0.0)	2.89	0.237
Shortness of breath	7 (3.4)	2 (3.6)	0 (0.0)	0.04	0.98
Earache	6 (2.9)	2 (3.6)	0 (0.0)	0.09	0.953
Throat dryness	5 (2.4)	1 (1.8)	0 (0.0)	0.1	0.949
Hoarseness of voice	6 (2.9)	0 (0.0)	0 (0.0)	1.69	0.428
Hematemesis	4 (1.9)	2 (3.6)	0 (0.0)	0.54	0.76
Mouth dryness	1 (0.5)	1 (0.8)	0 (0.0)		

Table 4 reveals that patients who did not show improvement following treatment had a significantly higher prevalence of halitosis (n=6, 20.7%) and snoring (n=3, 10.3%) as ENT symptoms (p <0.05). Additionally, for those patients whose symptoms worsened during the course of treatment, there was a notable prevalence of shortness of breath (SOB) affecting 25% of this group (p <0.05).

**Table 4:** Relationships between response to treatment and ENT symptoms (N=263)

Variable	Response to treatment			χ2	p value
	Getting worse, n (%)	Improvement, n (%)	No change, n (%)		
Dysphagia	1 (25)	73 (31.7)	9 (31)	0.08	0.958
Sore throat	1 (25)	61 (26.5)	12 (41.4)	2.83	0.243
Dysphonia	2 (50)	40 (17.4)	7 (24.1)	3.41	0.182
Cough	1 (25)	30 (13)	3 (10.3)	0.69	0.707
Chocking	0 (0.0)	27 (11.7)	3 (10.3)	0.57	0.751
Tinnitus	1 (25)	15 (6.5)	0 (0.0)	4.46	0.107
Vertigo	1 (25)	14 (6.1)	0 (0.0)	4.58	0.101
Odynophagia	0 (0.0)	12 (5.2)	3 (10.3)	1.5	0.471
Throat clearing	0 (0.0)	11 (4.8)	3 (10.3)	1.8	0.405
Halitosis	0 (0.0)	7 (3)	6 (20.7)	17.27	<0.001
Globus sensation	0 (0.0)	12 (5.2)	0 (0.0)	1.8	0.406
Snoring	1 (25)	6 (2.6)	3 (10.3)	9.2	0.01
Shortness of breath	1 (25)	8 (3.5)	0 (0.0)	6.66	0.036
Earache	0 (0.0)	8 (3.5)	0 (0.0)	1.18	0.553
Throat dryness	0 (0.0)	6 (2.6)	0 (0.0)	0.88	0.466
Hoarseness of voice	0 (0.0)	5 (2.2)	1 (3.4)	0.28	0.868
Hematemesis	0 (0.0)	5 (2.2)	1 (3.4)	0.28	0.868
Mouth dryness	0 (0.0)	2 (0.9)	0 (0.0)	0.28	0.865

## Discussion

This study aimed to explore the clinical characteristics of GERD in patients presenting with ENT symptoms who were subsequently referred for gastroenterology assessment at KFAFH in Jeddah, Saudi Arabia. The study also sought to evaluate the effectiveness of treatment and assess its implications for the overall management and prognosis of GERD in these patients. Among our cohort of 263 individuals referred for gastroenterology consultation due to their ENT symptoms, the average age was 50.71 years ± 20.22 years, and notably, 138 patients (52.4%) were 50 years or younger. The gender distribution revealed a significant predominance of female patients, comprising 168 individuals (63.9%). Moreover, nearly half of the participants (n=119, 45.2%) reported having chronic health conditions. Among these, diabetes mellitus was the most common, affecting 73 patients (61.3%), followed closely by hypertension, which was present in 68 patients (57.1%) (Table 1).

Endoscopic evaluations revealed that 61 patients (23.2%) exhibited abnormal findings, while a substantial majority (n=202, 76.8%) had normal endoscopic results. The duration of ENT symptoms varied, with 64 patients (24.3%) experiencing symptoms for a duration of one to two months, whereas 193 patients (73.3%) reported having symptoms for more than two months; the mean duration across the cohort was calculated to be 2.72 ± 1.15 months. Classification of GERD severity showed that the majority of patients (n=206, 78.3%) were categorized as LA grade A, while 56 patients (21.3%) were classified as LA grade B, and only one patient (0.4%) fell into the LA grade C category.

In terms of the specific ENT symptoms observed, the study identified dysphagia in 83 patients (31.6%), sore throat in 74 patients (28.1%), dysphonia in 18.6%, cough in 34 patients (12.9%), and a sensation of choking in 30 patients (11.4%). Furthermore, our analysis revealed that patients diagnosed with GERD LA grade C had a statistically significantly higher incidence of halitosis (p <0.05). However, no significant associations were observed between the classification of GERD and other ENT symptoms such as dysphagia, sore throat, or cough. When examining treatment responses, it was noted that patients who did not show improvement with treatment had a notably higher prevalence of halitosis (20.7%) and snoring (10.3%) compared to those who improved (p <0.05). Additionally, patients whose symptoms worsened were significantly more likely to report experiencing SOB as an ENT symptom, with a prevalence of 25% of the patients (p<0.05) (Table 4).

Our findings corroborate the growing body of evidence suggesting that diabetes mellitus plays a contributory role in the pathophysiology of GERD. Consistent with a prior meta-analysis, diabetes mellitus has been identified as a significant independent risk factor associated with an increased prevalence of both GERD and reflux esophagitis [25]. The extraesophageal symptoms documented in our study are consistent with those identified in earlier studies, which noted that GERD can often present with various ENT symptoms, including sore throat, dysphagia, and cough [16-18]. A particularly novel aspect of our research is the demonstration of a direct correlation between the severity of GERD and the presence of halitosis, a relationship that has not been extensively explored in previous studies. This underscores the importance of considering halitosis as a potential indicator of significant reflux activity, thereby enabling healthcare professionals to better identify patients who may require advanced medical intervention. Additionally, findings from endoscopic assessments and pH monitoring suggested the presence of abnormal findings in patients who presented with extraesophageal symptoms, even when results from PPI trials were insufficient to confirm a definitive diagnosis [25-27]. The data imply that relying solely on a single PPI trial is inadequate for confirming GERD, advocating for the adoption of more comprehensive diagnostic methods, such as 24-hour pH monitoring, as a standard practice for accurate GERD diagnosis and effective treatment planning.

Regarding therapeutic interventions, our study found that 203 patients (87.5%) showed improvement following the initiation of the PPI trial regimen. These results are consistent with previous studies that reported improvement rates of approximately 82% [28,29]. The underlying mechanism of action for PPIs involves the inhibition of the gastric H⁺-K⁺ ATPase enzyme, which leads to a significant reduction in gastric acid production. This reduction plays a crucial role in minimizing the reflux of acidic gastric contents into the upper aerodigestive tract. By decreasing acid levels, PPIs help alleviate direct mucosal irritation, edema, and inflammation of the pharynx and larynx. This process facilitates the healing of previously damaged tissues, ultimately leading to a reduction in symptoms. Moreover, by suppressing gastric acid secretion, PPIs elevate the pH level of the gastric mucosa. This change not only diminishes the corrosive effects of gastric acid but also inhibits the activation and enzymatic activity of pepsin, an important contributor to mucosal injury that can occur even at non-acidic pH levels. Thus, the therapeutic effects of PPIs extend beyond mere acid suppression, contributing to a comprehensive approach to managing GERD and its associated ENT symptoms [30].

Clinical implications

Research shows that patients who present with ENT symptoms must receive care through multidisciplinary approaches for proper GERD diagnosis and management. GERD identification among this patient demographic requires cooperation between gastroenterologists and otolaryngologists to deliver proper medical interventions. The diagnosis of GERD requires combined medical expertise since other diseases present similar symptoms, including infections and allergies, as well as malignancies. Our data reveals the need to avoid using PPI trials exclusively for diagnosis because such practices might produce incorrect diagnoses and improper treatments. Clinical staff need to focus on halitosis assessment among reflux disease patients since this symptom is strongly related to severe GERD. Further assessment by means of an esophageal manometer alongside pH monitoring can help establish reflux severity for better treatment planning. Snoring together with poor treatment response encourages doctors to consider employing combined pharmaceutical and nonpharmaceutical approaches, including lifestyle changes alongside surgical treatment options, for patients who are unresponsive to regular therapies.

Strengths and limitations

This study provides essential information regarding GERD clinical patterns among Saudi Arabian patients who receive medical care from ear, nose, and throat specialists because research in this area remains scarce in the Saudi Arabian territory. The relatively large sample size provides useful insight, although the retrospective design remains a limitation due to possible reporting biases while dealing with missing information. Our capacity to establish definitive links between ENT symptoms and GERD is restricted by the absence of a control group during the study. The study lacked control measures for potential confounding variables despite not considering factors such as diabetes mellitus and hypertension with GERD and its management.

Recommendations for future research

Future investigations need to adopt prospective study approaches involving extensive and diverse patient populations to prove current research findings while examining halitosis and additional varied symptoms for their significance as GERD indicators. An investigation of how diabetes and hypertension affect GERD diagnosis would provide essential knowledge for medical practitioners to improve their patient treatment plans. Comparative studies examining how well pH monitoring, endoscopy, and manometry diagnose GERD within ENT symptoms would establish better diagnostic procedures for this patient population.

## Conclusions

Our research highlights the significant challenges in diagnosing GERD in patients presenting with ENT symptoms, emphasizing the necessity for coordinated care among specialists. Evidence from the study suggests that GERD should be considered in cases where patients exhibit ENT symptoms such as dysphagia or sore throat, especially if these symptoms persist beyond initial medical treatments or for prolonged periods. The findings also highlight halitosis and snoring as potential indicators for identifying severe or treatment-resistant GERD, warranting further investigation into comprehensive disease management strategies. By focusing on accurate diagnostic methods for GERD and effective treatment options in patients referred from ENT practices, we can enhance healthcare outcomes and reduce unnecessary medical interventions. This coordinated approach is essential for improving patient care and achieving better therapeutic results in this population.
